# Population pharmacokinetics of lumefantrine in pregnant and non‐pregnant women with uncomplicated *Plasmodium falciparum* malaria in Western Kenya

**DOI:** 10.1111/bcp.70318

**Published:** 2025-10-28

**Authors:** Elizabeth Juma, Junjie Ding, Martin Ongas, Nelly Koskei, Kevin Onyango, Florence Oloo, Rashid Aman, Gilbert Kokwaro, Joel Tarning, Bernhards Ogutu

**Affiliations:** ^1^ Centre for Clinical Research Kenya Medical Research Institute Kisumu Kenya; ^2^ CREATES Strathmore University Nairobi Kenya; ^3^ Mahidol Oxford Tropical Medicine Research Unit, Faculty of Tropical Medicine Mahidol University Bangkok Thailand; ^4^ Centre for Tropical Medicine and Global Health, Nuffield Department of Medicine University of Oxford Oxford UK; ^5^ African Centre for Clinical Trials Nairobi Kenya; ^6^ Strathmore Business School Strathmore University Nairobi Kenya; ^7^ Infectious Diseases Data Observatory, Nuffield Department of Medicine University of Oxford Oxford UK

**Keywords:** lumefantrine, non‐linear mixed effect model, pharmacokinetics, *Plasmodium* falciparum malaria, pregnancy

## Abstract

**Aim:**

This study intends to assess the pharmacokinetic properties and treatment response of lumefantrine in pregnant and non‐pregnant women with uncomplicated *Plasmodium falciparum* malaria infection in Western Kenya.

**Methods:**

Seventy‐five women with uncomplicated *P*. *falciparum* malaria were enrolled, including 25 non‐pregnant, 30 pregnant women in the second trimester and 20 pregnant women in their third trimester. The participants received a standard dose of artemether–lumefantrine (80/480 mg) twice daily for 3 days. Densely venous plasma samples were collected. Nonlinear mixed‐effects modelling was used to characterize the pharmacokinetic properties of lumefantrine, and the effects of pregnancy was assessed on all pharmacokinetic parameters by a full covariate modelling approach.

**Results:**

The concentration‐time data of lumefantrine were described adequately by a two‐compartment disposition model, with a flexible transit absorption and first‐order elimination. Covariate modelling results demonstrated that pregnancy status or gestational age had a significant impact on both elimination clearance (CL) and the central volume of distribution (Vc) of lumefantrine. The estimated pregnancy effects on CL and Vc were 23% (95%CI: 10.8–34.8%) and 28% (95%CI: 7.3–51.8%), respectively. Pregnant women exhibited lower drug exposure compared to non‐pregnant women, with the geometric mean ratios (GMRs) of 0.76 (95% CI: 0.57–1.01), 0.79 (95% CI: 0.63–0.99) and 0.69 (95% CI: 0.51–0.94) for area under the concentration‐time curve (AUC), maxinum concentration (*C*_max_) and Day 7 concentration, respectively. Other covariates did not significantly affect the pharmacokinetics of lumefantrine. The 28‐day polymerase chain reaction (PCR)‐corrected parasitological cure was 100% for both pregnant and non‐pregnant women.

**Conclusions:**

The exposure to lumefantrine was lower in pregnant women, compared to non‐pregnant women, with uncomplicated *P. falciparum* infection. This lower drug exposure might increase the risk of treatment failure with artemether–lumefantrine in pregnant women, especially if susceptibility to either drug is reduced. Continuous assessment and monitoring of the efficacy of artemether–lumefantrine in pregnant women are warranted.

**What is already known about this subject?**

- WHO has recommended artemisinin‐based combination therapies for uncomplicated malaria in the second and third trimesters of pregnancy since 2006.
- In Kenya, artemether–lumefantrine is the recommended first line treatment for uncomplicated malaria in pregnancy.
- The pharmacokinetic properties of lumefantrine has been studied in clinical studies conducted across Asia and Africa, but inconsistent results have been reported on the impact of pregnancy on the pharmacokinetics of lumefantrine.

**What this study adds?**

- Pregnant women exhibited a significantly faster clearance and larger volume of distribution for lumefantrine, compared to non‐pregnant women, resulting in an approximately 30% lower drug exposure.
- This reduced drug exposure warrants continuous assessment and monitoring of the efficacy of artemether–lumefantrine in pregnant women, particularly in areas of reduced drug susceptibility.

## 1 INTRODUCTION

Malaria burden remains high with an estimated 463 million cases and 597 000 deaths in 2023.^1^ Pregnancy increases the vulnerability to malaria infection in all women living in areas of malaria risk because of suppressed immunity. Malaria in pregnancy is an important preventable cause of maternal and neonatal morbidity and mortality. In 2023, in 33 moderate‐to‐high transmission countries in the African Region, there were an estimated 36 million pregnancies of which 12.4 million (34%) were infected with *Plasmodium falciparum* malaria.^1^ It is estimated that exposure to malaria infections in pregnant women resulted in 901 000 neonates with low birthweight in 2023.^1^

The WHO has recommended artemisinin‐based combination therapies (ACTs) for uncomplicated malaria in the second and third trimesters of pregnancy since 2006.^2^ Artemether–lumefantrine was the first co‐formulated ACT developed and pre‐qualified by WHO, and is by far the most commonly used ACT today. Lumefantrine binds to haem within the infected red blood cells resulting in its accumulation and development of free radicals, both of which are toxic to the malaria parasite. In combination, artemether and lumefantrine have a rapid action against malaria parasites and produce a fast recovery from symptoms of malaria.^3^ A large phase 3 clinical trial demonstrated the efficacy and safety of the use of artemether–lumefantrine in African pregnant women with uncomplicated *P*. *falciparum* malaria.^4^ In Kenya, artemether–lumefantrine is the recommended first line treatment for uncomplicated malaria in all trimesters of pregnancy.^5^

Physiological changes during pregnancy, such as elevated liver enzyme activity, altered plasma protein level, and prolonged gastric emptying time may affect the absorption, distribution, metabolism and elimination (ADME) processes of drugs. Sub‐optimal drug concentrations in pregnant women could increase the risk of therapeutic failure and the development of drug‐resistant parasites. Lumefantrine is insoluble in water, and absorption is slow and variable and must be administered with a small amount of fat to maximize its absorption. Lumefantrine is primarily metabolized by the liver enzyme CYP3A4 into its active metabolite, desbutyl‐lumefantrine, which is present at very low concentrations compared to its parent compound.^6^ The pharmacokinetic (PK) characteristics of lumefantrine has been studied in several clinical studies across Asian and African countries. However, the findings regarding the impact of pregnancy on the PK of lumefantrine has been inconsistent in previous reports. Some studies showed substantially lower exposure to lumefantrine (e.g. day 7 concentration) in pregnant women,^7^, ^8^, ^9^ while other studies demonstrated no significant pregnancy effect on PK properties of lumefantrine.^10^, ^11^ This discrepancy has largely been attributed to variations in study design (such as PK sampling and sample size) and data analysis approaches employed (non‐compartmental analyses vs population PK analyses).

In the current study, we aimed to assess the PK properties of lumefantrine in pregnant and non‐pregnant women with uncomplicated malaria infections. The secondary objective was to evaluate the therapeutic response in both pregnant and non‐pregnant populations.

## 2 METHODS

### 2.1 Ethics statement

Ethical approval was provided by the Kenya Medical Research Institute Scientific and Ethics Review Unit and was registered with the Pan African Clinical Trial Registry (PACTR201211000451437).

### 2.2 Study participants

This PK study was conducted at Ahero County Hospital in Kisumu County in western Kenya, a hospital that serves a pre‐dominantly rural population in an area where malaria transmission is high and perennial. The objectives of the study were explained to potential study participants at the antenatal clinic and outpatient department of the health facility. The participants agreed and provided consent to join the study.

Fifty pregnant women aged 18–40 years in their second or third trimester of pregnancy and 25 non‐pregnant women presenting with uncomplicated *P*. *falciparum* malaria were eligible for enrolment. Eligibility criteria were fever ≥37.5°C or a history of fever in the last 24 h or other symptoms of malaria, mono‐infection with *P. falciparum* (parasite count between 1000 and 200 000 parasites/μL), haemoglobin ≥8 g/dL, absence of other co‐morbidities or contraindication to first line antimalarial treatment, and willingness to stay at the hospital for at least 3 days. Key exclusion criteria were a history of administration of any anti‐malarial drug or other antimicrobials with antimalarial properties in the month prior to presentation.

### 2.3 Treatment regimen

All study participants received directly observed treatment with four tablets of artemether/lumefantrine (AL) 20/120 mg tablets (Coartem®, Novartis AG), at hours 0, 8, 24, 36, 48 and 60. The study medicines were given orally with water, followed by 250 mL of milk or a light meal.

### 2.4 Clinical and laboratory assessment

A full medical history and physical examination was conducted. For pregnant women, additional antenatal tests not required for the study, such as HIV testing, were also undertaken. Gestational age was determined using the date of the last monthly period. Because ultrasonography was not undertaken routinely at this rural facility, only those with discrepant date and physical examination findings were referred to a referral hospital for a dating ultrasound.

Haemoglobin was measured as part of a complete blood count. Thick and thin blood films were stained with Giemsa. Parasite density of asexual *P. falciparum* parasites was determined by assuming a white blood cell count of 8000 per microlitre for the study population. Parasite and white cell counts were determined after an examination of at least 200 high‐powered microscope fields. A blood smear was classified as negative after scanning at least 100 high power fields without detecting any infected red blood cells. Parasite DNA was extracted from dried blood spots and analysed by real‐time polymerase chain reaction (PCR) to ascertain microscopically confirmed parasite clearance times and to distinguish recrudescent from new infections.

### 2.5 Follow‐up

All participants were admitted to hospital for 3 days for observed drug administration and then discharged. The participants were followed up for a total of 28 days. Clinical and parasitological evaluation and PK blood sampling were performed during hospitalization and follow‐up visits at the study site. Duplicate thick and thin blood films were prepared for parasitological outcomes, and filter paper spots were taken pre‐dose and every 8 h post first dose of artemether–lumefantrine until two consecutive slides were declared negative and thereafter during follow‐up on days 7, 14 and 28.

### 2.6 Pharmacokinetic sampling

An indwelling venous catheter was inserted into the forearm for PK sample collection and flushed after each blood draw with a heparinised saline solution. For the purpose of sampling and to minimize the number of blood draws, study participants in each of the three groups (non‐pregnant women, pregnant women in the second trimester and pregnant women in the third trimester) were randomly divided into two PK sampling arms. In the first arm, 2 mL of blood was drawn prior to dosing, and at 0.5, 1.5, 3, 6, 12, 30, 48 (pre‐dose 5), 60 (pre‐dose 6), 61, 64, and 68 h after the first dose of artemether–lumefantrine. In the second arm, the same amount of blood was drawn prior to dosing and then at 1, 2, 4, 8, 24 (pre‐dose 3), 42, 54, 60.5, 62, 66 and 72 h after the first dose of artemether–lumefantrine. Within each group, half of the participants were assigned to the first PK sampling arm and the other half to the second PK sampling arm.

For all participants (all groups and sampling arms), one blood sample was also drawn on all of the following days: 4, 5, 6, 7 and 14 after the first dose of artemether–lumefantrine. All samples were centrifuged immediately at 4°C 3000 *g* for 7 min (Universal 320R refrigerated centrifuge), and the plasma initially stored in liquid nitrogen on site before being transferred to a −80°C freezer at the Kenya Medical Research Institute laboratories in Kisumu.

### 2.7 Drug quantification

The PK plasma samples were shipped to and measured at the Clinical Pharmacology Laboratory, Mahidol Oxford Tropical Research Unit (MORU), Bangkok, Thailand. The plasma samples were quantified for lumefantrine using protein precipitation, followed by liquid chromatography mass spectrometry.^12^ Quality control samples at low, middle and high concentrations were analysed in triplicate within each analytical batch to ensure accuracy and precision during the analysis. The coefficients of variation during lumefantrine quantification (*n* = 18 at each concentration) were 6.10%, 4.69% and 2.50% at 25, 1600 and 3200 ng/mL, respectively. The lower limit of quantification (LLOQ) was set to 10 ng/mL for lumefantrine.

### 2.8 Population PK modelling approach

The population PK analysis was performed using NONMEM v. 7.4 (ICON Development Solutions, Ellicott City, MD, USA), compiled using gFortran (version 4.60). R studio (2024.09.1 Build 394) was used to construct the output visualization. Perl‐speaks NONMEM (PsN; version 4.6.0) and Pirana (Version 23.10.1) were used for automation and diagnostics during the model‐building process. Discrimination between models during the model building phase was based on standard visual diagnostics and the objective function value (OFV), calculated as proportional to twice the log‐likelihood of the data. A drop in OFV (∆OFV) of 6.64 and 10.8 was considered statistically significant at *p* < .01 and *p* < .001, respectively, between two hierarchical models after inclusion of one additional parameter (one degree of freedom difference). Potential systematic errors, model misspecification and overall predictive performance of the final population PK model was assessed using goodness‐of‐fit diagnostics and simulation‐based diagnostics (visual predictive checks; *n* = 1000 simulations). The percentage of eta shrinkage was calculated to evaluate the reliability of the empirical Bayes estimates. The sampling importance resampling (SIR) approach^13^ was used to evaluate parameter uncertainty. The median and the 2.5th–97.5th percentiles of the SIR estimates were reported as a measure of model precision and robustness of the final model.

The first‐order conditional estimation method with *η*–*ε* interaction (FOCE‐I) was used during the model‐building procedure (except for M3 which requires a Laplacian estimation method). Various methods were considered to investigate the influence of censored PK concentration data (i.e. drug concentrations measured below the LLOQ),^14^ including M1 (omitting LLOQ data), M6 (imputing the first concentration below the LLOQ as half of the LLOQ and omitting subsequent LLOQ data for an individual), M3 (maximizing the likelihood of predicting censored data below the LLOQ) and the M7 + approach (inflation of the residual error).^15^ These methods were evaluated by comparing the proportion of predicted and observed concentration below the LLOQ, using categorical visual predictive checks.

### 2.9 Structure model

One‐, two‐ and three‐compartment disposition models were evaluated to describe the concertation‐time profile of lumefantrine. A flexible transit‐absorption model, with a stepwise addition of a fixed number of 1–10 transit compartments, was used to describe the absorption of lumefantrine. Relative bioavailability (*F*) was set to unity for the population but included to allow variability to be estimated between patients in the absorption of lumefantrine. A Box–Cox transformed distribution was also evaluated for this parameter.

### 2.10 Random effect model

Inter‐individual variability was added exponentially to all PK parameters (Equation 1)

(Eq 1) $$\theta_{i}=\theta \times \exp\left(\eta_{i,\theta }\right)$$

where, *θ*_*i*_ is the individual parameter estimate, *θ* is the population value of the investigated parameter and *η*_*i,θ*_ is the inter‐individual variability, assumed to be normally distributed with a zero mean and variance *ω^2^*.Inter‐occasion variability (IOV) between different dose occasions on absorption parameters (i.e. bioavailability and mean transit time) was also considered. Few PK samples were available from second to fifth dose, and dose occasions were therefore pooled together and evaluated as two separate dose occasions (i.e. first to third dose, and fourth to sixth dose). Residual unexplained variability was modelled as additive, proportional, or combined additive and proportional errors on log‐transformed concentrations.

### 2.11 Covariate model

Body weight was added as an allometric function on all clearance and volume parameters a priori, considering the strong biological prior of this relationship.^8^ The allometric function was centred on a typical body weight of 70 kg and scaled with an exponent of 0.75 and 1 for clearance and volume parameters, respectively (Equations 2 and 3).

(2) $$CL_{i}=CL\times \exp\left(\eta_{i,CL}\right)\times {\left(\frac{{\mathrm{BW}}_{i}}{{\mathrm{BW}}_{\text{median}}}\right)}^{0.75},$$

(3) $$V_{i}=V\times \exp\left(\eta_{i,V}\right)\times {\left(\frac{{\mathrm{BW}}_{i}}{{\mathrm{BW}}_{\text{median}}}\right)}^{1.0},$$

where ${\mathrm{BW}}_{i}$ is individual body weight and BW median is the median body weight of the population.

Lumefantrine has been reported to show a dose‐limited absorption, and this was unconditionally added on relative bioavailability as described in Equation (4). The saturation parameter, dose_50_ (3.84 mg/kg), was not estimated but taken from a large pooled analysis of lumefantrine and refers to the dosage at which a typical patient reaches 50% saturation of the absorption.^8^

(4) $$F=100\%\times \left(1-\frac{\text{Dose}}{\text{Dose}+{\text{Dose}}_{50}}\right).$$

Pregnancy or trimester as a proportional categorical covariate, and gestational age as a continuous covariate were evaluated on all PK parameters using a backward elimination approach by incorporating pregnancy on all parameters followed by a formal stepwise elimination (*p* < .05). Moreover, pregnancy effects were also evaluated by a full covariate approach. This was accomplished by implementing pregnancy as a covariate on key primary PK parameters (elimination clearance, central volume of distribution, mean transit absorption time and relative bioavailability) followed by fitting this full covariate model to 500 bootstrapped data sets, and evaluating the effect of pregnancy on both primary and secondary PK parameters (area under the concentration‐time curve [AUC], maximun concentration [*C*_max_] and day 7 concentration). In each bootstrap run, secondary PK parameters were directly outputted from NONMEM, and descriptive results were summarized across the 500 runs. Because of identifiability issues, pregnancy could not be evaluated on all parameters in the model with a full covariate approach, and the parameters above was deemed most appropriate on account of their impact on overall exposure and peak concentrations. Prolonged gastric emptying is a well‐known physiological alteration associated with pregnancy. Thus, mean transit absorption time was judged as a more appropriate parameter to describe this delay in absorption rather than *K*_a_. Therefore, *K*_a_ was not included in the full covariate model assessment.

Lastly, other covariates, such as age, baseline temperature and baseline parasitaemia were evaluated on all PK parameters in a stepwise manner with a forward selection (*p* = .05, *df* = 1, ∆OFV = 3.84) and a stricter backward elimination (*p* = .01, *df* = 1, ∆OFV = 6.63).

### 2.12 Nomenclature of targets and ligands

Key protein targets and ligands in this article are hyperlinked to corresponding entries in http://www.guidetopharmacology.org, and are permanently archived in the Concise Guide to PHARMACOLOGY 2021/2022.^16^

## 3 RESULTS

### 3.1 Patient characteristics

Between August 2013 and April 2014, 50 pregnant women with uncomplicated *P. falciparum* malaria, 30 in the second and 20 in the third trimesters, were enrolled in the study. Twenty‐five non‐pregnant women with uncomplicated *P. falciparum* malaria were also enrolled. The demographic and medical characteristics of the study participants are shown in Table 1. Pregnant women tended to be younger than their non‐pregnant controls and had significantly lower peripheral blood parasite densities. They were also more likely to have no fever, but there were no differences in bodyweight and height between the two groups. However, bodyweight in pregnant women in the third trimester [median 66 (range: 51–8 kg] was higher compared to non‐pregnant women [median 62 (range: 40–87)] and pregnant women in the second trimester [median 57 (range: 40–74) kg].

**TABLE 1:** Characteristics of the study population at enrolment.

Parameter	Pregnant women *N* = 50	Non‐pregnant women *N* = 25	p
Age, years	20.2 (18.0–35.3)	25.2 (18.1–35.6)	<0.001
Bodyweight, kg	62.0 (40.0–86.6)	59.5 (45.0–89.0)	0.986
Height, cm	164 (145–184)	165 (150–176)	0.920
Temperature, °C	36.8 (35.6–39.4)	37.6 (36.3–39.4)	0.004
Parasite density, count/mL	10 960 (1000‐199 360)	24 320 (2560‐152 960)	0.022
Gestational age, weeks	26(13–40)	‐	‐
Day 14 PCR corrected cure rate	45/45 (100%)	25/25 (100%)	‐
Day 28 PCR corrected cure rate	44/44 (100%)	22/22 (100%)	‐

*Note*: Continuous data are presented as median (min–max), and cure rates are presented as number of patients (%).

#### 3.1.1 Treatment outcomes

Of the 50 pregnant women enrolled in study, two withdrew consent to continue participation on day 0 after first dose of medication while four were lost to follow‐up after day 3. Three of the 25 non‐pregnant women were lost to follow‐up on day 28. Forty‐four and 22 pregnant and non‐pregnant women, respectively, completed 28 days of follow‐up and were eligible for per protocol analysis for treatment outcomes. Table 1 shows PCR corrected therapeutic outcomes on days 14 and 28. The day 28 cure rate for artemether–lumefantrine was 100% in each group. There were three recurrences of *P. falciparum* infection during follow‐up, two pregnant and one non‐pregnant woman (all on day 28), and all of which were confirmed by PCR to be re‐infections (not recrudescent malaria). The parasite clearance half‐life between pregnant and non‐pregnant women were comparable (3.24 vs 2.87 h, *p* = .06). AL was found to be efficacious and well tolerated in this population with no serious adverse events reported.

#### 3.1.2 Population PK modelling

A total 1151 venous lumefantrine plasma concentrations were collected from 50 pregnant and 25 non‐pregnant women from whom at least one post dosing sample was drawn. Eighty‐six plasma samples (7.4%) were reported to be below the LLOQ. However, a good agreement was observed between predicted and observed data below the LLOQ when omitting these samples from the analysis (M1 approach), and other censoring methods (M3, M6 and M7+) were not further investigated. Thus, LLOQ samples were omitted in the PK model building.

**FIGURE 1:**
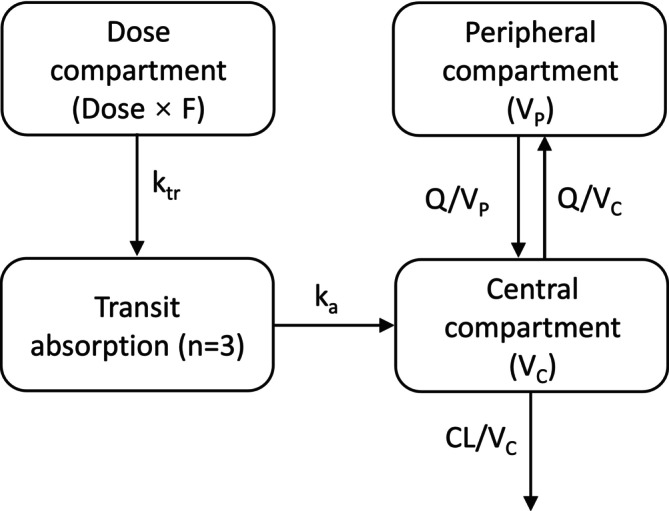
**Graphical overview of the PK model structural of lumefantrine.** *k*_tr_ is the absorption transit rate constant. *K*_a_ is the first order absorption rate constant. CL is the elimination clearance. *V*_C_ is the central volume of distribution. *V*_P_ is the peripheral volume of distribution. *Q* is the inter‐compartment clearance. *F* is the relative oral bioavailability.

The lumefantrine concentration‐time profile was described adequately by a two‐compartment disposition model with first‐order elimination. A three‐compartment disposition model did not result in a further improvement in model fit (*p* > 0.05). A flexible transit‐absorption model (optimal number of compartments = 3) with separately estimated transit and absorption rate constants improved model fit significantly compared to the first‐order absorption model (*p* < .001; ∆OFV = − 359). The model structural is shown in Figure 1.

Applying a Box–Cox transformation of inter‐individual variability of relative bioavailability improved model fit further (∆AIC = −14.9). A combination of a proportional and additive error showed the best residual diagnostics and was implemented throughout in the model development.

Implementation of bodyweight on all clearance and volume parameters showed a small improvement in model fit (*p* > .05, ∆OFV = −1.75) and was retained because of the strong biological prior of this covariate. A dose‐dependent effect on the relative bioavailability was incorporated into the model a priori and showed a borderline significant improvement in model fit (*p* < .05, ∆OFV = −3.95).

**FIGURE 2:**
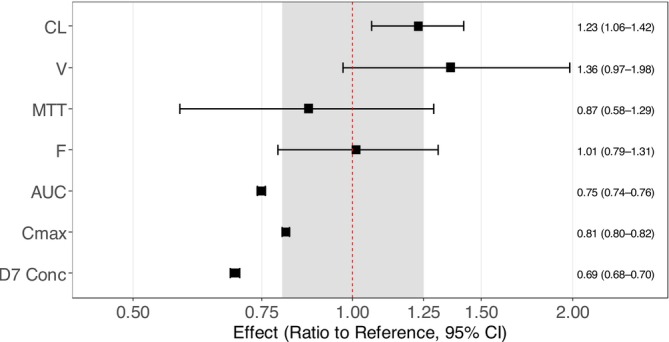
**Forest plot of the pregnancy effect on PK parameters of lumefantrine using a full covariate modelling approach.** The covariate effects were derived from 500 bootstrap runs. MTT is the mean transit time. CL is the elimination clearance. *F* is the relative oral bioavailability. *V*_C_ is the central volume of distribution. AUC is the area under the concentration‐time curve from time zero to infinite. *C*_max_ is the maximum concentration. D7 Conc is the day 7 concentration. The grey shaded area represents a 0.8–1.25 range.

Implementation of pregnancy on all PK parameters, followed by a stepwise backward elimination, demonstrated that pregnancy status was a significant (∆OFV = − 8.27 and −8.28, respectively, *p* < .01) covariate on clearance and central volume of distribution (Vc), respectively. Gestational age was also identified as a significant covariate on clearance and central volume parameters (*p* < .01 and .05, respectively). On the contrary, trimester did not show a significant impact on any PK parameters. The model with pregnancy status (categorical covariate) had the lowest OFV and was chosen as the final model. This finding was further confirmed by a full covariate model approach, which showed similar covariate effects resulting in a significant impact of pregnancy on clearance and central volume of distribution, while absorption rate and relative bioavailability were not affected (Figure 2 **)**. Additionally, the secondary PK parameters AUC, *C*_max_ and day 7 concentration, derived from the full covariate approach, were lower in pregnant women compared to non‐pregnant women (Figure 2).

Other covariates, such as baseline parasitaemia and age, did not have significant impact on the PK parameters of lumefantrine. Inter‐occasion variability was added on the absorption rate constant (ΔOFV = −45.1) and relative bioavailability (ΔOFV = −54.3), resulting in a significantly superior.

**FIGURE 3:**
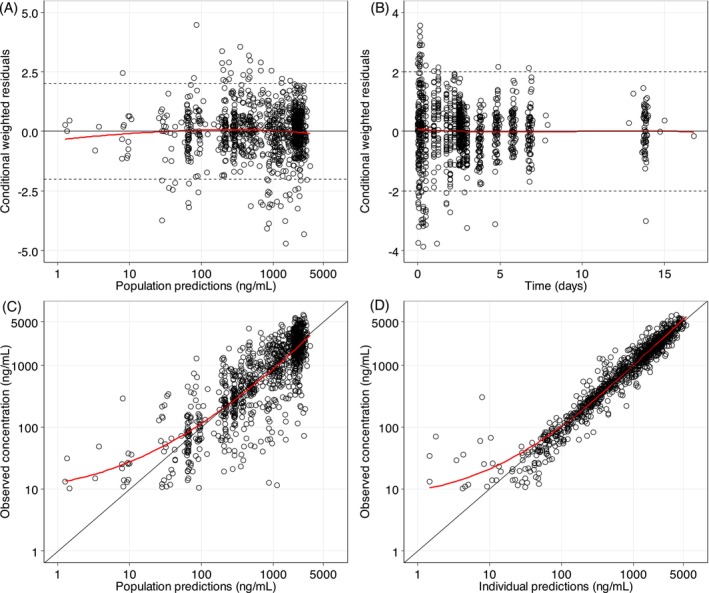
**Goodness‐of‐fit plots for the final population PK model of lumefantrine.** Conditional weighted residuals versus population predictions (A) and time (B). Observed plasma concentrations versus population predictions (C) and against individual predictions (D). The solid black lines are the identity lines, and the solid red lines are the locally weighted least squares regression line, the dashed lines in Figure 3A and B are the ±2 limit of conditional weighted residual.

**FIGURE 4:**
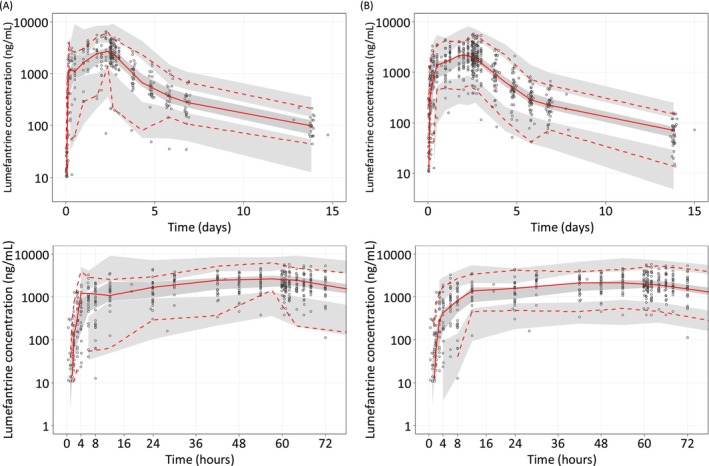
**Visual predictive check of the final population PK model for lumefantrine in** (A) **non‐pregnant and** (B) **pregnant women based on 2000 stochastic simulations.** Open circles represent the observations, and solid lines represent the 5^th^, 50^th^ and 95^th^ percentiles of the observed data. The shaded areas represent the 95% confidence intervals around the simulated 5^th^, 50^th^ and 95^th^ percentiles. The bottom panels show the predictive performance during the first 72 h of treatment.

**FIGURE 5:**
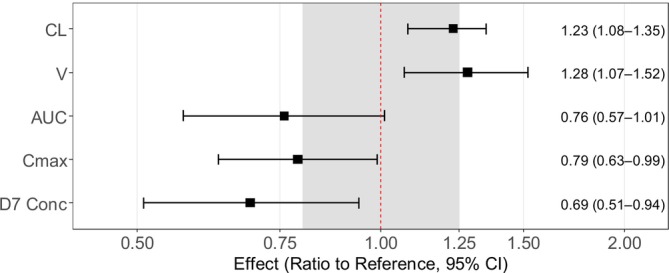
**Forest plot of the pregnancy effect on PK parameters of the final model.** The reference population was non‐pregnant women. The covariate effect of pregnancy on secondary PK parameters was expressed as geometric mean ratio (GMR) for pregnant versus non‐pregnant women. CL is the elimination clearance. *V*_C_ is the central volume of distribution. AUC is the area under the concentration‐time curve from time zero to infinite. *C*_max_ is the maximum concentration. D7 Conc is the day 7 concentration. The grey shaded area represents a 0.8–1.25 range.

As shown in the goodness of fit (Figure 3), the developed final population PK model described the observed concentration‐time data well, with no obvious model misspecifications or errors, and the model showed a high predictive performance (Figure 4). Final parameter estimates were precise with small relative standard errors (Table 2). Table 3 presents the secondary PK parameters derived from the final population PK model, comparing pregnant and non‐pregnant women. The pregnancy effect on PK parameters in the final model are shown in Figure 5. Pregnant women exhibited a 23.2% (95%CI: 10.8–34.8%) and 28.1% (95%CI: 7.3–51.8%) higher CL and V, respectively. Pregnant women exhibited lower drug exposure compared to non‐pregnant women, with a geometric mean ratio (GMR) of 0.76 (95%CI: 0.57–1.01), 0.79 (95%CI: 0.63–0.99) and 0.69 (95%CI: 0.51–0.94) for AUC, *C*_max_ and day 7 concentration, respectively.

**TABLE 2:** Final parameter estimates of lumefantrine population PK model in pregnant and non‐pregnant women.

Parameter	NONMEM	SIR median (95%CI)	Shrinkage (%)
	Population estimates (%RSE)		
*Population parameters*			
MTT (h)	4.70 (6.9)	4.62 (4.01–5.28)	
No. transit comp.	3 (fixed)	‐	
*K*_a_ (1/h)	0.411 (12.7)	0.401 (0.322–0.533)	
*F* (%)	100 (fixed)	‐	
Box‐cox on F	−0.690 (13.2)	−0.664 (−0.830 to −0.463)	
CL/*F* (L/h)	4.03 (5.1)	4.03 (3.66–4.45)	
*V*_C_/*F* (L)	108 (8.6)	108 (90.3–127)	
Q/*F* (L/h)	1.67 (7.1)	1.67 (1.46–1.93)	
*V*_p_/*F* (L)	184 (6.1)	185 (168–211)	
*Inter‐individual or inter‐occasion variability (%RSE)*			
MTT (IIV)	74.4 (9.5)	75.5 (63.1–89.5)	16.7
*K*_a_ (IOV)	109 (11.6)	111 (85.0–135)	60.4
*F* (IOV)	64.0 (6.9)	64.1 (54.6–72.8)	16.5
*V*_C_/*F* (IIV)	14.1 (41.9)	14.6 (3.7–25.4)	54.8
*Unexplained residual error*			
Proportional (%)	33.0 (3.4)	32.9 (31.0–35.2)	
Additive (ng/mL)	12.0 (26.3)	12.5 (8.6–18.9)	
*Covariate relationships*			
Dose_50_ (mg/kg) on *F*	3.84 (fixed)	‐	
Pregnancy on CL (%)	23.2 (25.8)	23.3 (10.8–34.8)	
Pregnancy on *V*_c_ (%)	28.1 (38.8)	27.0 (7.3–51.8)	

*Note*: Population estimates are given for a ‘typical’ non‐pregnant woman weighting 70 kg with acute *P*. *falciparum* malaria infection. MTT is the mean transit time. *K*_a_ is the absorption rate constant. CL/*F* is the elimination clearance. *V*_C_/*F* is the central volume of distribution. *Q*/*F* is the inter‐compartmental clearance. *V*_p_/*F* is the peripheral volume of distribution. *F* is the relative oral bioavailability. Pregnancy status was implemented using a proportional covariate model $\theta =\theta_{\mathrm{TV}}\cdot \left(1+\theta \mathrm{cov}\right)$, where $\theta_{\mathrm{TV}}$ is the typical value of a given parameter, and θcov is the categorical pregnancy effect compared to a non‐pregnant reference population. Dose_50_ was implemented using a saturation model on *F* [1 − (Dose/[θ + Dose])]. Coefficients of variation for inter‐individual variability (IIV) and inter‐occasion variability (IOV), presented in the table, were calculated as 100 × (e^variance^)^1/2^. SIR: sampling importance resampling. Relative standard errors (%RSE) were derived from SIR estimates.

**TABLE 3:** Lumefantrine population PK model‐derived secondary parameters in pregnant and non‐pregnant women.

Secondary parameters	Non‐pregnancy	Pregnancy with second trimester	Pregnancy with third trimester
*T*_1/2_ (h)	109 (103–120)	103 (96.8–108)	106 (99.8–111)
*C*_max_ (μg/mL)	3.21 (1.02–5.31)	2.28 (0.99–4.82)	2.44 (0.904–3.98)
AUC (h·μg/mL)	275 (80.7–452)	184 (42.7–446)	208 (57.6–361)
Day 7 concentration (ng/mL)	277 (84.5–525)	182 (32.8–478)	192 (48.3–410)

*Note*: *T*_1/2_ is the terminal elimination half‐life. AUC is the area under the concentration‐time curve from time zero to infinite. *C*_max_ is the maximum plasma concentration after dose. Secondary‐parameter estimates are derived from the Bayesian post hoc estimates. The data are presented as median (95% CI).

## 4 DISCUSSION

The current analysis indicated that both pregnancy status and gestational age were a significant covariate on PK parameters of lumefantrine. PK exposures were approximately 30% lower in pregnant women compared to that in non‐pregnant women. This reduced PK exposure suggests a potential risk of treatment failure with artemether–lumefantrine in pregnant women with uncomplicated *P*. *falciparum* malaria, especially in areas with reduced susceptibility to any of the two drugs.

Artemether–lumefantrine has been used as the first line treatment for pregnant women in the second and third trimesters in East Africa for more than a decade and currently allowed in the first trimester as well. The clinical question regarding PK alternation during pregnancy is crucial to inform the dose in this underserved population. Clinical PK studies of lumefantrine in pregnant women have been conducted in a South East Asian population^17^, ^18^ and African population.^7^, ^9^, ^10^, ^11^ However, the results have been inconsistent. Kloprogge et al. reported that the inter‐compartmental clearance of lumefantrine was 37% lower in Uganda pregnant women (*n* = 26) compared with non‐pregnant women (*n* = 17), resulting in 27% lower day 7 concentrations in pregnant women.^7^ A study with sparse PK collection showed a 34% lower relative bioavailability of lumefantrine in Tanzania pregnant women (*n* = 33) compared to non‐pregnant women (*n* = 22).^9^ In contrast, another two PK studies indicated no significant effect of pregnancy on PK exposure of lumefantrine.^10^, ^11^ Additionally, a pooled PK analysis based on 3938 patients, including 123 pregnant women, indicated an increased absorption rate during pregnancy, resulting in 20% lower day 7 concentrations compared with non‐pregnant adults.^8^

There remains limited PK data on lumefantrine in sub‐Saharan African patients, where the medicine is most needed. In this study, we included 50 pregnant women and 25 non‐pregnant women living in an area of high malaria transmission to help address the PK data gap of lumefantrine in this region. In the current analysis, using a backward elimination approach found that pregnancy status or gestational age significantly increased both the elimination clearance and central volume of lumefantrine, and these pregnancy effects were further confirmed by a full covariate modelling approach. The pregnancy‐associated covariate effects of pregnancy resulted in a 25% lower AUC, 19% lower *C*_max_ and 31% lower day 7 concentration in pregnant women. The PK exposure was comparable between the second and third trimester of pregnancy. Notably, lower day 7 concentrations have been consistently observed across the majority of published studies in pregnant women.^7^, ^8^, ^9^, ^10^ However, this finding was not clearly interpreted in previous studies. According to the current analysis, it is reasonable to conclude that the lower day 7 concentrations in pregnant women is because of the observed increase in elimination clearance.

It has been reported that pregnancy is associated with increased enzyme activity of specific CYP enzymes, leading to alterations in the PK properties of various drugs.^19^ Lumefantrine is metabolized primarily by CYP3A4 enzyme in the liver into its main metabolite, desbutyl‐lumefantrine, which has substantially lower PK concentrations compared to the parent drug. It has been reported that hepatic CYP3A activity is consistently increased (35%–38%) during all stages of pregnancy.^20^ However, no change has been observed in intestinal CYP3A activity or expression during pregnancy.^21^ In the present modelling analysis, we demonstrated a 23% increase in elimination clearance during the second and third trimester of pregnancy, which aligns with the reported changes of CYP3A activity during pregnancy. Moreover, pregnancy is associated with changes in the volume of distribution as a direct result of increased blood volume (50%), changes in plasma proteins, tissue binding and body weight. Lumefantrine mainly binds to high density lipoproteins (HDL) in plasma with negligible binding to plasma albumin or α1‐acid glycoprotein.^22^ However, HDL shows no major change during pregnancy.^23^ This suggests that the change in distribution because of protein binding during pregnancy is unlikely. The 28% increase in central volume of distribution of lumefantrine identified in this study is most likely attributed to the increased blood volume during pregnancy. Furthermore, renal elimination of lumefantrine is minimal.^24^ Taken together, we conclude that the approximately 30% lower exposure to lumefantrine and the reduced peak concentrations during pregnancy is most likely a result from increased CYP3A activity and blood volume.

Additionally, parasitaemia were not significantly correlated with any PK parameters of lumefantrine in this study. This finding was inconsistent with a large published individual patient data analysis, which demonstrated that the relative bioavailability of lumefantrine decreased with increasing enrolment parasitaemia.^8^ This discrepancy may be attributed to the narrow distribution of baseline parasite density in our study.

In this study, no treatment failures were observed during the 28‐day follow‐up period in this small cohort of pregnant women. Additionally, a large clinical trial has demonstrated that artemether–lumefantrine is still efficacious and safe for treating pregnant women with uncomplicated malaria.^4^ In our study, we found that lumefantrine day 7 concentrations were 30% lower in pregnant women, with 44% of individuals falling below the 200 ng/mL threshold, previously associated with increased risk of therapeutic failure, compared to only 20% of non‐pregnant women. With the emergence of artemisinin resistance in several sub‐Saharan African countries^25^, ^26^ and indications of reduced lumefantrine susceptibility^27^ in some areas, this reduced drug exposure might increase the risk of treatment failure with artemether–lumefantrine in pregnant women. These findings highlight the need for continuous assessment and monitoring of the drug's efficacy in this population.

Our study was limited by not being able to model the lumefantrine active metabolite desbutyl‐lumefatrine. However, lumefantrine is the major contributor to therapeutic efficacy and, it is not likely that altered desbutyl‐lumefatrine exposures would have a substantial effect on the therapeutic outcome of this combination therapy. Moreover, prolonged gastric emptying and gastrointestinal transit times are commonly seen in pregnant women.^28^ However, this study appears to be under‐powered to detect any pregnancy‐associated effects on gastrointestinal parameters. Moreover, the unbalanced sample size in control group (half of that in pregnant groups), and differences in baseline parameters, such as age, parasitaemia and temperature, also limit the comparability between groups.

In conclusion, exposure to lumefantrine was approximately 30% lower in pregnant women with uncomplicated *P. falciparum* infection in Western Kenya compared to non‐pregnant women. This reduced exposure might increase the risk of treatment failure, especially in areas of emerging drug resistance. Continuous assessment and monitoring of the efficacy of artemether–lumefantrine in pregnant women are necessary.

## AUTHOR CONTRIBUTIONS

Junjie Ding, Elizabeth Juma and Joel Tarning wrote the article. Junjie Ding and Joel Tarning conducted the pharmacometrics modelling. Bernhards Ogutu and Elizabeth Juma designed the research. Bernhards Ogutu, Elizabeth Juma and Kevin Onyango performed the research. All authors reviewed the final manuscript.

## CONFLICT OF INTEREST STATEMENT

The authors declare no conflicts of interest.

## Supporting information


**Data S1.** NONMEM code for the final population PK model of lumefantrine in pregnant and non‐pregnant women.

## Data Availability

The data supporting the findings of this study are available from the corresponding author upon reasonable request.
