# Supplementary material for: Population pharmacokinetics of lumefantrine in pregnant and non‐pregnant women with uncomplicated Plasmodium falciparum malaria in Western Kenya
Source: Br J Clin Pharmacol. 2025 Oct 28;92(3):911–21. doi: 10.1111/bcp.70318 (PMC12930023; doi:10.1111/bcp.70318)
Supplement: Supplementary file 1 — Data S1. NONMEM code for the final population PK model of lumefantrine in pregnant and non‐pregnant women. [file BCP-92-911-s001.docx]

**NONMEM code for the final population PK model of lumefantrine in pregnant and non-pregnant women**

$INPUT ID TIME DV CMT AMT DOSE MDV EVID OCC AGE BQL PREG GA WT

$DATA NM_LF.csv

$SUBROUTINE

ADVAN5 TRANS1

$MODEL

COMP = (1) ; Dose

COMP = (2) ; Central compartment

COMP = (3) ; Peripheral compartment

COMP = (4) ; Transit compartment 1

COMP = (5) ; Transit compartment 2

COMP = (6) ; Transit compartment 3

$PK

F1COVD = 1 - (DOSE / (THETA(9) + DOSE)) ; Dose covariate

CLCOVP = ( 1 + THETA(10)*PREG) ; Pregnancy covariate on clearance

V2COVP = ( 1 + THETA(11)*PREG) ; Pregnancy covariate on central volume

IF (OCC.EQ.1) IOVF1= ETA(8) ; IOV on F1

IF (OCC.EQ.2) IOVF1= ETA(9) ; IOV on F1

IF (OCC.EQ.1) IOVKA= ETA(11) ; IOV on KA

IF (OCC.EQ.2) IOVKA= ETA(12) ; IOV on KA

TVCL = THETA (1) * ((WT/70) ** 0.75)* CLCOVP ; Population clearance

CL = TVCL * EXP(ETA (1)) ; Individual clearance

TVV2 = THETA (2) * (WT/70) * V2COVP ; Population central volume

V2 = TVV2 * EXP(ETA (2)) ; Individual central volume

TVMTT = THETA (3) ; Population mean transit time

MTT = TVMTT * EXP(ETA (3)) ; Individual mean transit time

TVQ = THETA (4) * ((WT/70) ** 0.75) ; Population inter-compartment clearance 1

Q = TVQ * EXP(ETA (4)) ; Individual inter-compartment clearance 1

TVV3 = THETA (5) * (WT/70) ; Population peripheral volume 1

V3 = TVV3 * EXP(ETA (5)) ; Individual peripheral volume 1

SHP = THETA(6) * EXP(ETA(6)) ; Shape parameter for cox-box transformation

TETA = (EXP(ETA(7) + IOVF1)**SHP-1)/SHP ; Box- cox transformation

TVF1= THETA (7) * F1COVD ; Population relative bioavailability

F1 = TVF1 * EXP(TETA) ; Individual relative bioavailability

TVKA = THETA(8) ; Population absorption rate constant

KA = TVKA * EXP(ETA(10) + IOVKA) ; Individual absorption rate constant

NN = 3 ; Number of transit compartments

KTR = (NN+1)/MTT ; Transit rate constant

K14 = KTR ; Transit rate constant (COMP 1 --> 4)

K45 = KTR ; Transit rate constant (COMP 4 --> 5)

K56 = KTR ; Transit rate constant (COMP 5 --> 6)

K62 = KA ; Absorption rate constant (COMP 6--> 2)

K20 = CL/V2 ; Elimination rate constant (COMP 2 --> 0)

K23 = Q/V2 ; Distribution rate constant (COMP 2 --> 3)

K32 = Q/V3 ; Distribution rate constant (COMP 3 --> 2)

$ERROR

W = SQRT(SIGMA(1,1) + SIGMA(2,2)/EXP(IPRED)**2) ; Residual error

IF (F.GT.0) IPRED = LOG(F) ; Natural logarithm of predictions

IRES = IPRED-DV ; Individual residual error

IWRES = IRES/W ; Individually weighted residual error

Y = IPRED + EPS(1) + EPS(2)/EXP(IPRED) ; Additive and proportional residual error

$THETA

(0, 4.02) ; 1. Clearance

(0, 108) ; 2. Central volume

(0, 4.7) FIX ; 3. Mean transit time

(0, 1.67) ; 4. Inter-compartment clearance

(0, 184) ; 5. Peripheral volume

(-1, -0.692) ; 6. Shape parameter

(1) FIX ; 7. Relative bioavailability

(0, 0.412) ; 8. Absorption rate constant

(3.84) FIX ; 9. Dose covariate on relative bioavailability

(0, 0.235) ; 10. Pregnancy covariate on clearance

(0, 0.284) ; 11. Pregnancy covariate on central volume

$OMEGA

0 FIX ; 1. IIV clearance

0.0196 ; 2. IIV central volume

0.553 ; 3. IIV mean transit time

0 FIX ; 4. IIV inter-compartment clearance

0 FIX ; 5. IIV peripheral volume

0 FIX ; 6. IIV shape parameter

$OMEGA 0 FIX ; 7. IIV relative bioavailability

$OMEGA BLOCK(1) 0.411 ; 8. IOV relative bioavailability

$OMEGA BLOCK(1) SAME ; 9. IOV relative bioavailability

$OMEGA 0 FIX ; ; 10. IIV absorption rate constant

$OMEGA BLOCK(1) 1.19 ; 11. IOV absorption rate constant

$OMEGA BLOCK(1) SAME ; 12. IOV absorption rate constant

$SIGMA

0.109 ; 1. Proportional residual error

145 ; 2. Additive residual error

$ESTIMATE MAXEVAL=9990 METHOD=COND INTE NOABORT PRINT=1 POSTHOC
